# Lemon extract supported green synthesis of bimetallic CuO/Ag nanoporous materials for sensitive detection of vitamin D3

**DOI:** 10.1038/s41598-023-46774-w

**Published:** 2023-11-22

**Authors:** Gowhar A. Naikoo, Fay M. Almashali, Fatima A. S. Habis, Mustri Bano, Jahangir Ahmad Rather, Israr U. Hassan, Rayees Ahmad Sheikh, Palanisamy Kannan, Iman M. Alfagih, Murtaza M. Tambuwala

**Affiliations:** 1 Department of Mathematics and Sciences, College of Arts and Applied Sciences, Dhofar University, Salalah, PC 211 Oman; 2 Department of Chemical Engineering, College of Engineering, Dhofar University, Salalah, PC 211, Oman; 3 Department of Chemistry, Govt. Degree College Pulwama, Kashmir, 192301 India; 4 College of Biological, Chemical Sciences and Engineering, Jiaxing University, Jiaxing, 314001 People’s Republic of China; 5 Department of Pharmaceutics, College of Pharmacy, King Saud University, 4545 Riyadh, Saudi Arabia; 6 Lincoln Medical School - Universities of Nottingham and Lincoln, University of Lincoln, Brayford Pool, Lincoln Lincolnshire, LN6 7TS UK

**Keywords:** Molecular medicine, Nanoscience and technology

## Abstract

In modern era, deficiency of Vitamin D3 is predominantly due to limited exposure to sunlight and UV radiation resulting from indoor lifestyles. Several studies have revealed that vitamin D deficiency can lead to chronic vascular inflammation, diabetes mellitus, hypertension, congestive left ventricular hypertrophy, and heart failure. This study introduces a green synthesis of novel bimetallic nanoporous composite, CuO/Ag using lemon extract. The synthesized nanoporous material, CuO/Ag@lemon extract was characterized using several analytical techniques, including X-ray diffraction (XRD), transmission electron microscopy (TEM), scanning electron microscopy (SEM), and energy-dispersive X-ray spectroscopy (EDX). The CuO/Ag@lemon extract nanoparticles were immobilized on glassy carbon electrode (GCE) to prepare modified CuO/Ag@lemon extract–GCE interface. The electrocatalytic and electrochemical properties investigation was carried out on the modified electrode. using cyclic voltammetry (CV), differential pulse voltammetry (DPV), and amperometry for detecting of Vitamin D3. The DPV method displayed a linear response range of 0.02–22.5 µM with a detection limit of 2.62 nM, while the amperometric method showed a broader linear range of 0.25–23.25 µM with a detection limit of 2.70 nM with 82% modified electrode stability. The designed electrode exhibited a positive response to the inclusion of Vitamin D3 with electro-oxidation, reaching steady-state within 3.4 s, with 87% reproducibility within a day. The proposed method offers a rapid and sensitive platform for detection of Vitamin D3 with minimal interference from other molecules. The early diagnosis of Vitamin D3 deficiency using modified electrodes allows for early treatment, thereby preventing severe health complications.

## Introduction

In recent days, BMNPs are esteemed for their exclusive and splendid optical, magnetic, electronic^1–3^, and catalytic properties because of the coalescence of two distinct metal nanoparticles in an unambiguous pattern^4,5^. Their enactment often exceeds over their single counterparts endorsed to synergistic effects of combined metal nanoparticles^6–9^.

The modern need is to develop novel BMNPs having higher aspect ratio and surface plasmon band and use them to improve sensors with better selectivity, sensitivity, stability, and reliability^10–13^. The synthesis of BMNPs with controlled structures shows electrocatalytic properties and hence are paramount important in the field of sensor technology^14^. The tuning of metal nano-surfaces in BMNPs upon introduction of second metal nanoparticles are categorized into three main effects viz. ensemble effect, ligand effect and strain effect^15^. These three effects can be considered as vital for improving the electrocatalytic properties of BMNPs by increasing energy surface sites on modified electrode which resulted in improving charge transfer kinetics at the electrode − electrolyte interfaces^16^.

Traditionally BMNPs are synthesized by metallurgical methods which involves melting of two types of bulk metals under the proper circumstances^17^. These solid-state methods are operated at high-temperatures and annealing process usually takes long periods^18^. However, green technology offers a new opportunity for synthesis of BMNPs as it is an ecologically conscious, meek, unwavering, prompt, and economical method^19^. Typically, the production of BMNPs entails combining two distinct metal solutions in an aqueous medium, along with a reducing agent that is derived from plant extracts, ensuring an environmentally conscious approach^20^. During the process of synthesizing metallic nanoparticles, phytochemicals serve dual functions: firstly, as a reducing agent, and secondly, as a stabilizing agent for the nanoparticles^21,22^. Plant-mediated synthesis of BMNPs involves use of plant extract that is rich in reducing agents (flavonoids, terpenoids, and phenolic acid) which makes synthesized BMNPs more stable and varied in size and shape^23^. Karthik et al.^24–26^ offer a novel method for green production of silver nanoparticles (AgNPs) utilizing different leaf extract. This ecologically friendly technology eliminates the need of hazardous chemicals while demonstrating the promise of AgNPs in critical applications.

A review of the literature uncovered that lemon juice has been effectively employed for the synthesis and in-situ stabilization of silver and gold nanoparticles in aqueous environments^27,28^. These research highlight the importance of novel electrocatalytic materials and their many applications. Notably, several materials have showed outstanding catalytic characteristics in pharmaceutical analysis, helping to accurately determine individual medications. Furthermore, sophisticated nanocomposite materials have proven to be extremely effective at detecting environmental pollutants, particularly in the context of environmental monitoring. Furthermore, the impact of distinct doping techniques on materials has been investigated in the context of tackling issues in numerous scientific fields^29–32^. In this communication, we have used lemon juice rich in antioxidants viz., polyphenols, limonoids, ascorbic, and citric acid having potential to decrease ions possessing high oxidation states. We assume that an analogous mechanism may also works for the exploration of BM CuO/Ag nanoporous materials by utilizing lemon juice as both capping and reducing agent.

In recent times, vitamin D has gained significant attention in modern society, as individuals face challenges in obtaining the necessary amount of this essential vitamin for optimal health due to various socio-economic and environmental factors. Vitamin D is an indispensable fat-soluble vitamin for deciding bone health by sustaining the concentration levels of phosphorus and calcium in the body^33^. Vitamin D deficiencies can cause serious health problems including hypertension, Parkinson’s, Alzheimer’s, autoimmune diseases, neuropsychiatric disorders cardiovascular and cancer diseases^34^. Vitamin D exits in two main forms ergo calciferol (Vitamin D2) and cholecalciferol (Vitamin D3). Among two metabolic forms, the detection of Vitamin D3 form is preferred over vitamin D2, because meta-analysis shows that Vitamin D3 is more effectual at raising serum 25(OH)D concentrations than vitamin D2^35^.

The unadventurous techniques used for identifying Vitamin-D concentration are mass spectrometry, enzyme-linked immunosorbent assay, radioimmunoassay, and chromatography etc^36–38^. Though above procedures show high exactitude and sensitivity but they need trained operators, centralized laboratories, a lengthy procedure for sample preparations, costly equipment, which hinders their applications for the on-site detection^39^. The demand for development of low cost, affordable, highly selective and highly sensitive electrochemical sensor for monitoring of vitamin D is emergent in the field of sensor technology. The designing of an electrochemical sensor by suitable modification of the electrode surface can remarkably improve the sensor response for the detection of target molecules^40^.

**Figure 1:**
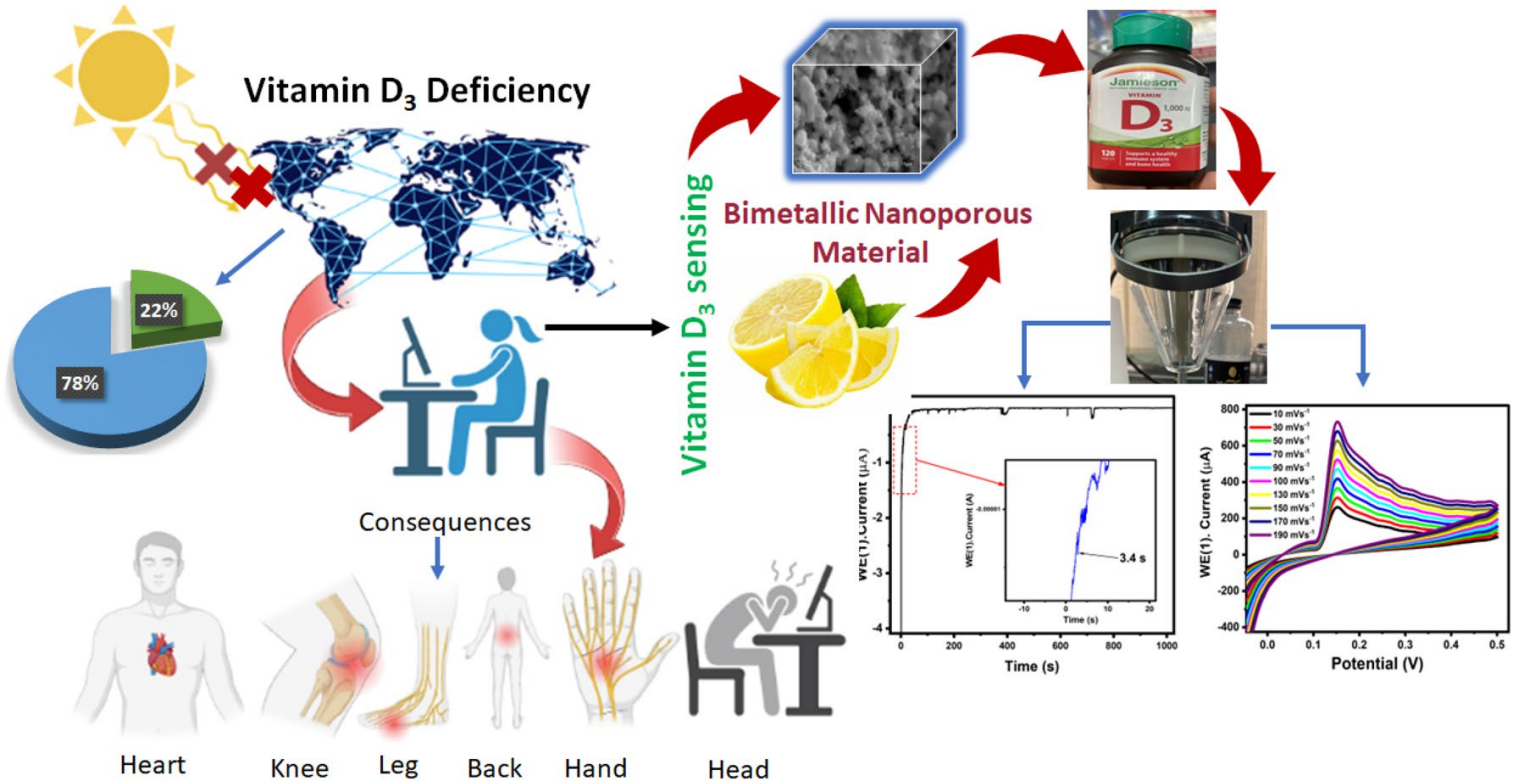
Bimetallic CuO/Ag@lemon extract-GCE has shown excellent responses towards vitamin D_3_.

In this perspective, current work designates green exploration of well-controlled CuO/Ag BMNPs structuralized by n-hexane and lemon extract and immobilized these synthesized BMNPs for uncovering of Vitamin D3 (Fig. 1). The synthesized CuO/Ag@n-hexane and CuO/Ag@lemon BMNPs were characterized by XRD, EDX, SEM and TEM techniques. The electrocatalytic performance of fabricated interfaces CuO/Ag@n-hexene-GCE and CuO/Ag@lemon extract-GCE was examined using CV, DPV, and amperometry for detection of Vitamin D3. The proposed method offers a rapid and sensitive platform for estimation of Vitamin D3 with minimal interference from other molecules. The early diagnosis of Vitamin D3 deficiency using modified electrodes allows for early treatment, thereby preventing severe health complications.

## Methods

### Synthesis of CuO/Ag@n-hexane and CuO/Ag@lemon extract nanoporous materials

**Figure 2:**
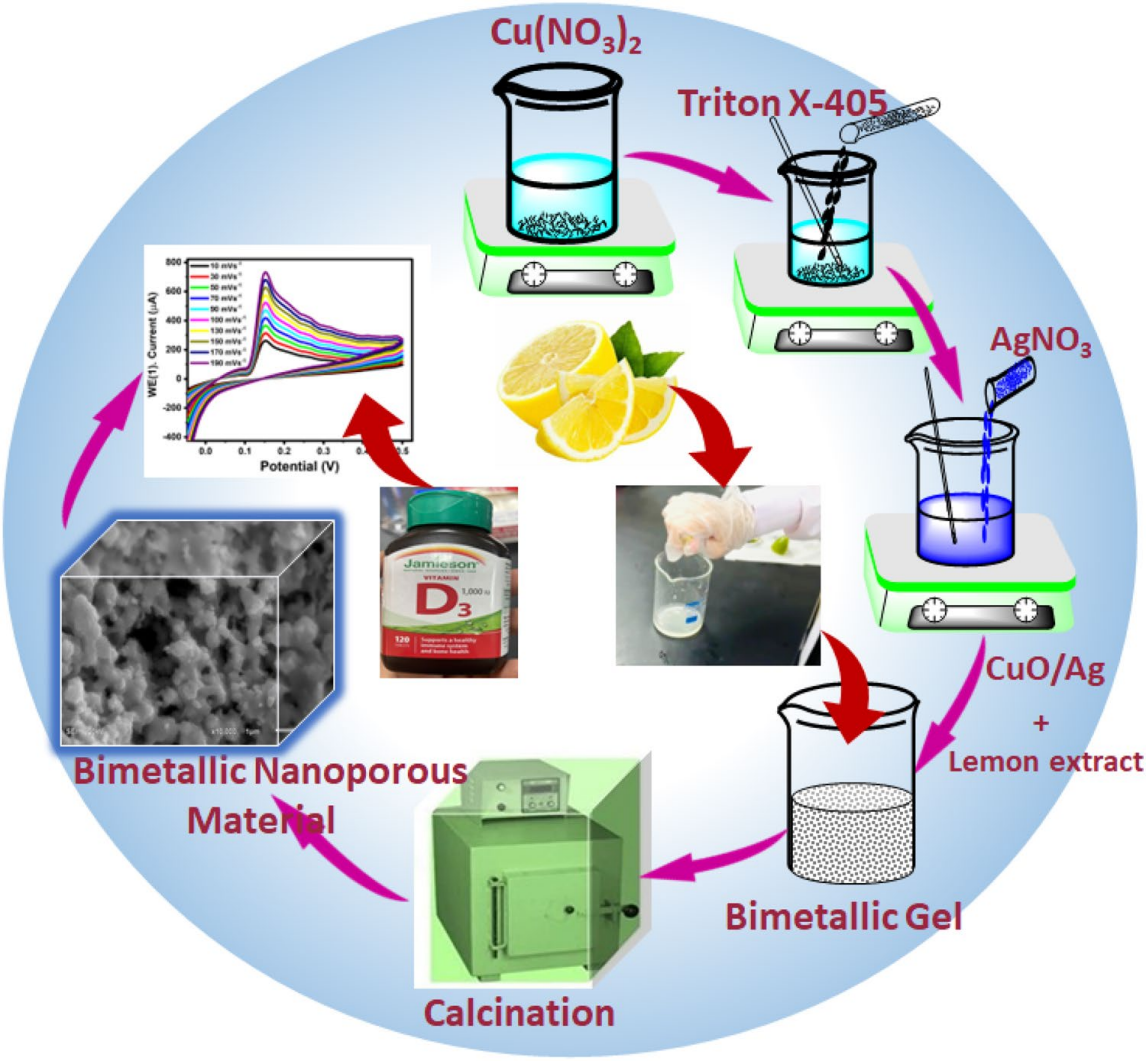
Synthetic representation of Bimetallic CuO/Ag nanoporous materials using lemon extract.

To synthesize CuO/Ag@n-hexene, a solution was prepared by dissolving 2 g of Cu(NO_3_)_2_ (50 wt% of, BDH) in 2 g of ultrapure water (50 wt%). To this solution, 4 g of triton X405 (Sigma-Aldrich, 70 wt %) were added dropwise along with 2 g of AgNO_3_ (57.14 wt%, Sigma-Aldrich) dissolved in 2 g of ultrapure water. Subsequently, 4 ml of n-hexene was added, and the resulting mixture was stirred for 30 min to form a gel that turned dark blue in color. To synthesize CuO/Ag@lemon extract, a solution was prepared by dissolving 2 g of Cu(NO_3_)_2_ (50 wt% of, BDH) in 2 g of ultrapure water (50 wt%). To this solution, 4 g of triton X405 (Sigma-Aldrich, 70 wt %) were added dropwise along with 2 g of AgNO_3_ (57.14 wt%, Sigma-Aldrich) dissolved in 2 g of ultrapure water. Subsequently, 4 ml of lemon extract (Citrus limon was collected from the lulu hypermarket, Salalah, Oman and it was cut into eight pieces without peeling (screw type) and the extract was obtained by using lemon squeezer and collected in a 100 ml beaker. The obtained lemon extract was filtered and its pH was recorded) was added, and the resulting mixture was stirred for 30 min to form a gel that turned dark blue in color. 'All the methods are in accordance with relevant guidelines'. Both the gel was then stored at room temperature for 120 h before being subjected to calcination for 5 h at 500 °C in a furnace, with a heating/cooling rate of 4.17 °C/min until it reached room temperature. The synthesized CuO/Ag@lemon extract nanoporous materials is characterized by various analytical techniques and later applied for detection of Vitamin D3 summarized in Fig. 2.

### Preparation of modified CuO/Ag@lemon extract–GCE sensing platform

All electrochemical experiments were conducted under room temperature conditions using a three-electrode system. This system included a Pt counter electrode, an Ag/AgCl reference electrode, and a glassy carbon working electrode (GCE) with a diameter of 3.0 mm. The working electrodes were prepared by modifying the surface of the unmodified glassy carbon electrode (b-GCE) with a dispersion of CuO/Ag@n-hexene and CuO/Ag@lemon extract nanoporous materials. The preparation of this dispersion involved sonicating 5.0 mg of the synthesized monoliths separately in ethanol (10 mL). Prior to the modification of the electrode, the GCE surface was polished using alumina slurry, cleaned with ethanol via sonication for a duration of 10 min, rinsed with distilled water, and then dried at room temperature. A 10 μL volume of the prepared dispersion was applied onto the GCE surface, and the solvent was allowed to naturally evaporate at room temperature. To optimize the performance of the electrodes, the modified GCEs (CuO/Ag@n-hexene-GCE and CuO/Ag@lemon extract-GCE) underwent electrochemical activation by subjecting them to cyclic potential sweeps ranging from -1.0 to + 2.0 V in a 0.1 M HNO_3_ solution until steady voltammograms were obtained. Following each electrochemical study, the modified electrode surfaces were cleansed with a 1.0 mM NaOH solution by performing potential sweeps in the opposite direction (from 1.0 V to 0.0 V).

### Instrumentation

The synthesized CuO/Ag@n-hexene and CuO/Ag@lemon extract nanoporous composites were characterized by many analytical techniques. Powdered X-ray Diffraction (P-XRD) analysis was conducted using X Pert PRO X-ray diffraction with Cu Ka radiation. Morphological studies (SEM and EDX) was performed using JEOL JSM-6510LA electron microscope. The structural details, size distribution, and morphology of nanoparticles were conducted by TEM analysis using JEM1400 instrument. The Muffle Furnace, MODEL: HD150 PAD was employed for the synthesis of all nanoporous materials. Electroanalytical (CV DPV and amperometry) studies were performed using Autolab PGSTAT204 FRA32M.

## Results and discussion

### X-ray diffraction (XRD) analysis

**Figure 3:**
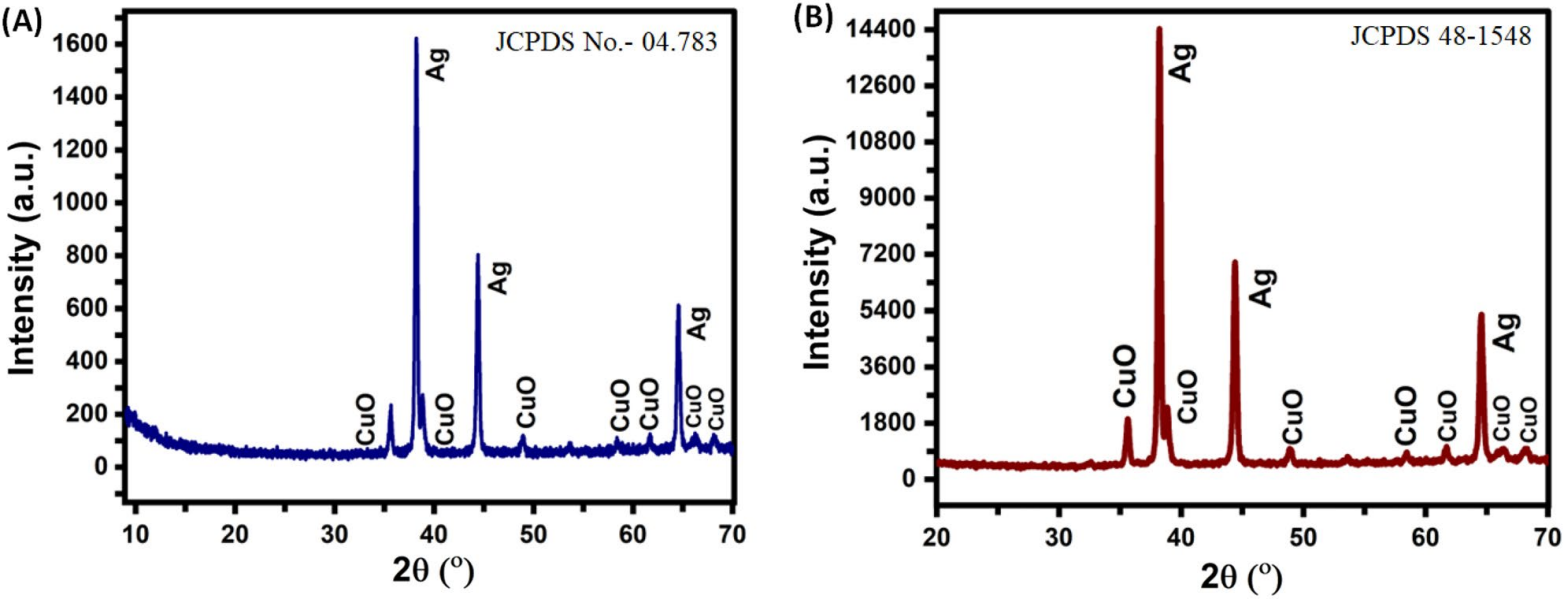
XRD patterns of synthesized (**A**) CuO/Ag@n-hexane and (**B**) CuO/Ag@lemon extract nanoporous materials.

Figure 3 (A & B) depicts the X-ray diffraction (XRD) patterns CuO/Ag@n-hexane and CuO/Ag@lemon extract monoliths. The calcined samples of CuO/Ag@n-hexane (Fig. 3A) showed diffraction peaks at 35.60°, 38.17°, 38.89°, 48.92°, 58.47°, 61.70°, 66.37°, and 68.21°, with corresponding d-spacing values of 2.5197 Å, 2.3552 Å, 2.3136 Å, 1.8601 Å, 1.5786 Å, 1.5027 Å, 1.4045 Å, and 1.3736 Å. These peaks attributed to [110], [022], [200], [202], [002], [202], [222], and [022] lattice planes of copper oxide [JCPDS 48–1548]. In addition, XRD pattern of CuO/Ag@n-hexane monolith shows peaks at 38.28°, 44.33°, and 64.55°, with corresponding d-spacing values of 2.3488 Å, 2.0414 Å, and 1.4427 Å, attributed to [211], [200], and [311] lattice planes, of silver [JCPDS No.- 04.783]. The presence of these peaks indicates that bimetallic monolith has a face-centered cubic (FCC) crystal structure due to the presence of silver.

Figure 3B shows similar XRD peaks of synthesized CuO/Ag@lemon extract with a slight shift in their positions compared to CuO/Ag@n-hexane nanoporous material. Moreover, the presence of a structural directing agent (SDA) led to an increase in the intensity of diffraction peaks in XRD pattern of CuO/Ag@lemon extract compared to CuO/Ag@n-hexane. The variations in intensity are caused by differences in the scattering and absorption properties of the materials involved. CuO/Ag@lemon is expected to have greater scattering and absorption capabilities than hexane due to the addition of nanoparticles or other geometrical features. The reason for formation of monoliths can be attributed to the combined effect of surfactant and solvent used during synthesis process. The surfactant acts as a stabilizing agent, while solvent controls morphology of monoliths. XRD Studies confirms both types of synthesized monoliths exhibited crystalline behaviour.

### Morphological characterization of synthesized CuO/Ag@n-hexane and CuO/Ag@lemon extract nonporous materials

**Figure 4:**
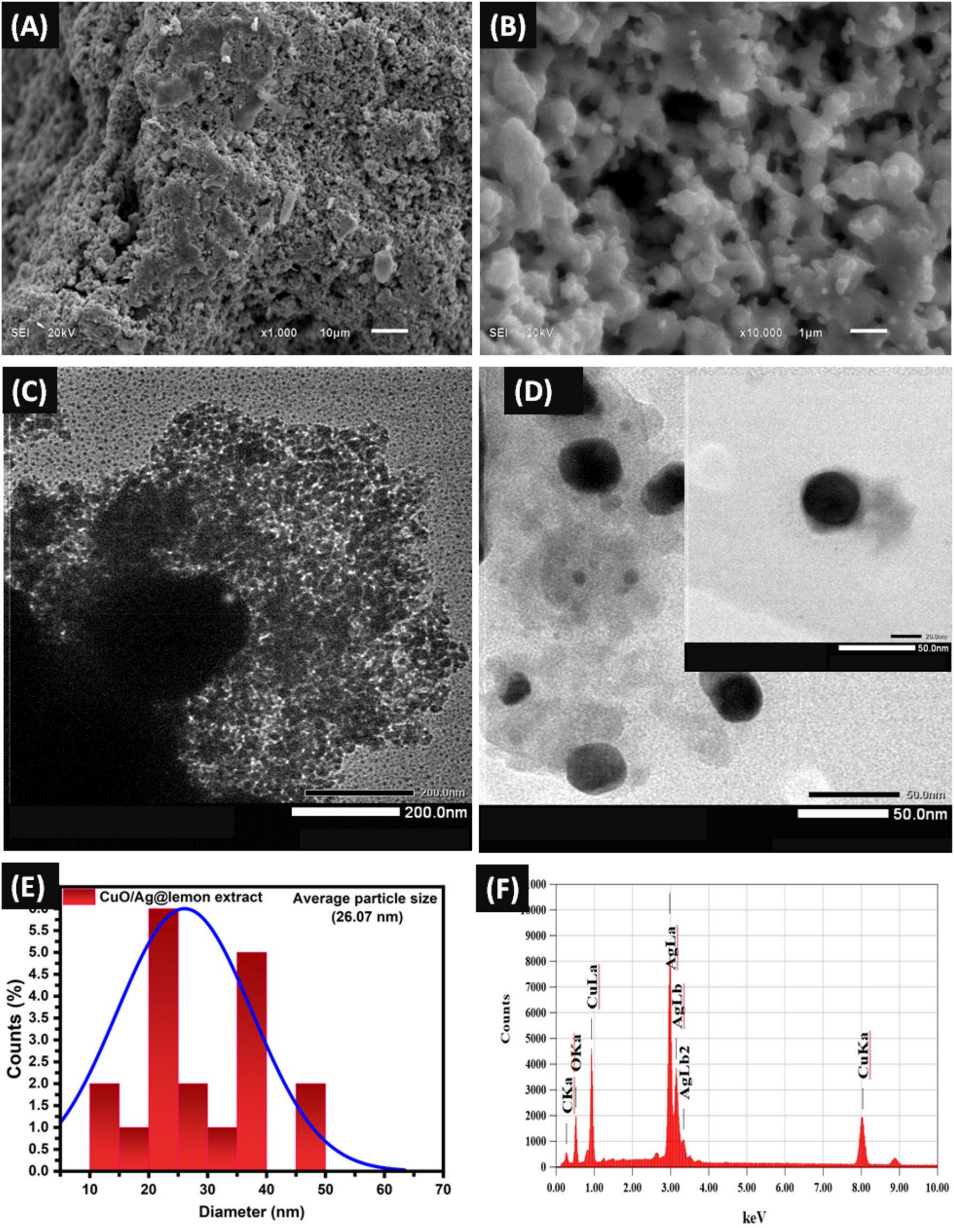
SEM Images of (**A**) CuO/Ag@n-hexane nonporous BMNPs at 10 µm (**B**) CuO/Ag@lemon extract nonporous material at 1 µm. TEM images of (**C**) CuO/Ag@n-hexane nonporous material at 100.0 nm scales and (**D**) CuO/Ag@lemon extract nonporous material at 50.0 nm (inset single particle TEM at 20.0 nm). (**E**) CuO/Ag@lemon extract nanoporous material diameter distribution. (**F**) EDX of bimetallic CuO/Ag@lemon extract nonporous material.

The SEM (Fig. 4A,B) study of synthesized BMNPs CuO/Ag structuralized by n-hexane and lemon extract obtained at 10 μm and 1 μm scale shows bimetallic nanoparticles are stabilized by covalent bonds between copper, silver, and oxygen atoms and in addition the presence of n-hexane and lemon extract acts as structural directing agents. Structural directing agents (SDAs) help direct growth of bimetallic nanoparticles and maintain their specific structure or shape. In present studies, n-hexane and lemon extract as SDAs ensure CuO/Ag BMNPs maintain their bimetallic structure and do not undergo unwanted reactions. The non-polar solvent n-hexane produces less crystalline bimetallic nanoparticles due to weaker stabilization and less effective structural directing properties. In contrast, a polar lemon extract containing organic compounds leads to more clear and uniform nanoparticle formation through stronger stabilization and more effective structural directing properties. These observations suggest that the choice of solvent and extract can significantly impact the properties and formation of synthesized bimetallic nanoparticles. The crosslinking between Cu and Ag, indicating that there may be metal–metal bonds present in the bimetallic nanoparticles. These bonds contribute to the stability and unique properties of the BMNPs and the role of n-hexane and lemon extract as structural directing agents helps to ensure that these bonds are maintained.

Figure 4C of CuO/Ag@n-hexane nanoporous material at 200.0 nm scale reveals a porous structure with nanoparticles dispersed throughout. The pores are likely formed due to the removal of n-hexane during the synthesis process. The nanoparticles appear to be agglomerated, indicating weaker stabilization and less effective structural directing by n-hexane. Figure 4D of CuO/Ag@lemon extract nanoporous composite at 50.0 nm scale reveals a similar porous structure, but with more uniform and clear nanoparticle formation, as observed in the inset single particle image at 20.0 nm scale. This suggests that the use of lemon extract as a polar solvent and structural directing agent results in better stabilization and more effective structural directing of the nanoparticles.

Figure 4E shows the diameter distribution of CuO/Ag@lemon extract nanoporous material, indicating a relatively narrow size distribution. This is likely due to the ability of the organic compounds in the lemon extract to act as surfactants or as a capping agent, controlling the size and dispersity of the nanoparticles during the synthesis process. Figure 4F shows the EDX analysis of CuO/Ag@lemon extract nanoporous material, confirming the presence of silver, copper, and oxygen elements in the bimetallic nanoparticles. The relative abundance of each element suggests that the synthesis process has been successful in producing bimetallic nanoparticles with desired elemental compositions.

### Electroanalytical determination of Vitamin D3 at CuO/Ag@lemon extract-GCE interface

**Figure 5:**
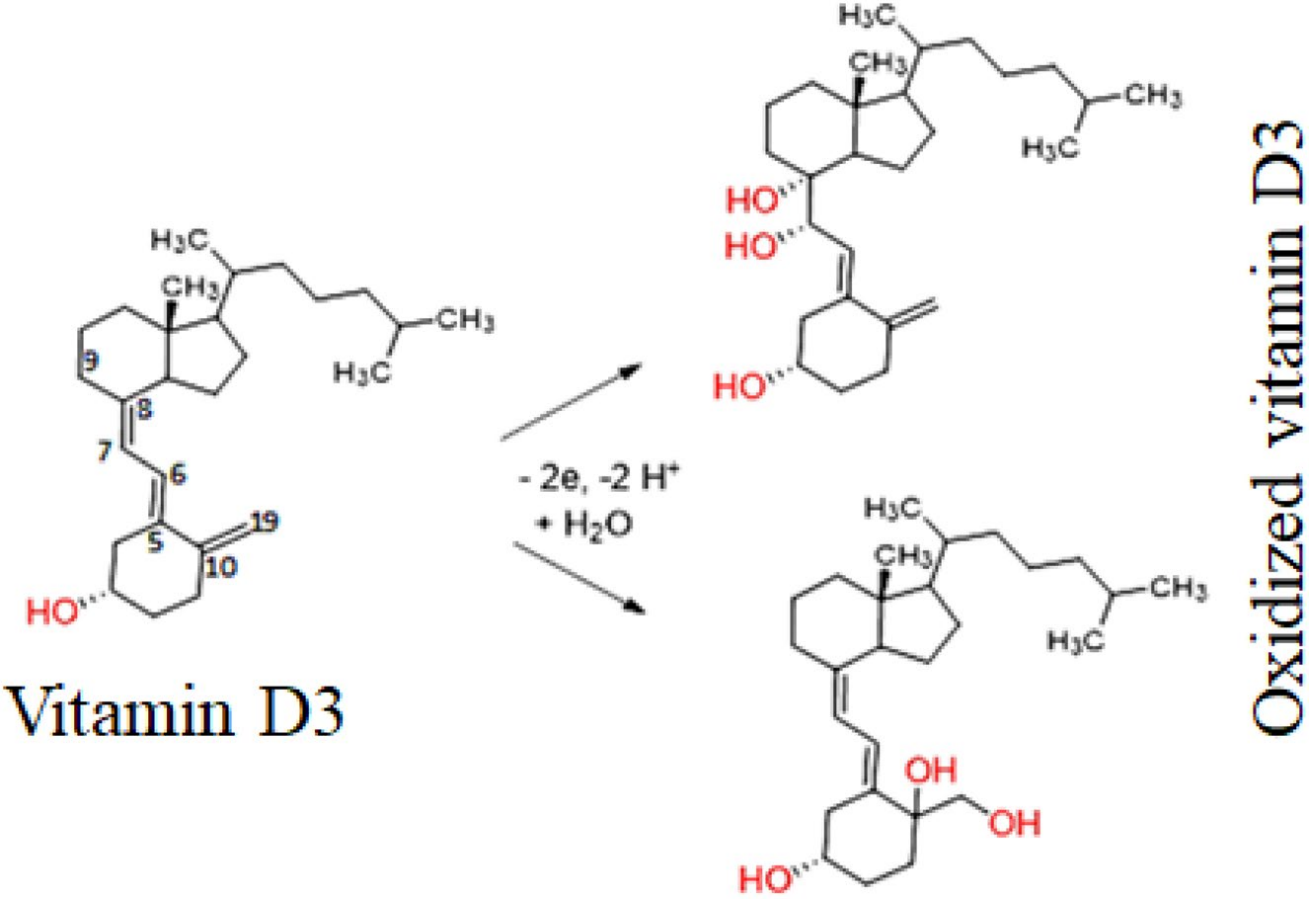
Mechanism of Electrooxidation of Vitamin D3 at CuO/Ag@lemon extract − GCE interface.

**Figure 6:**
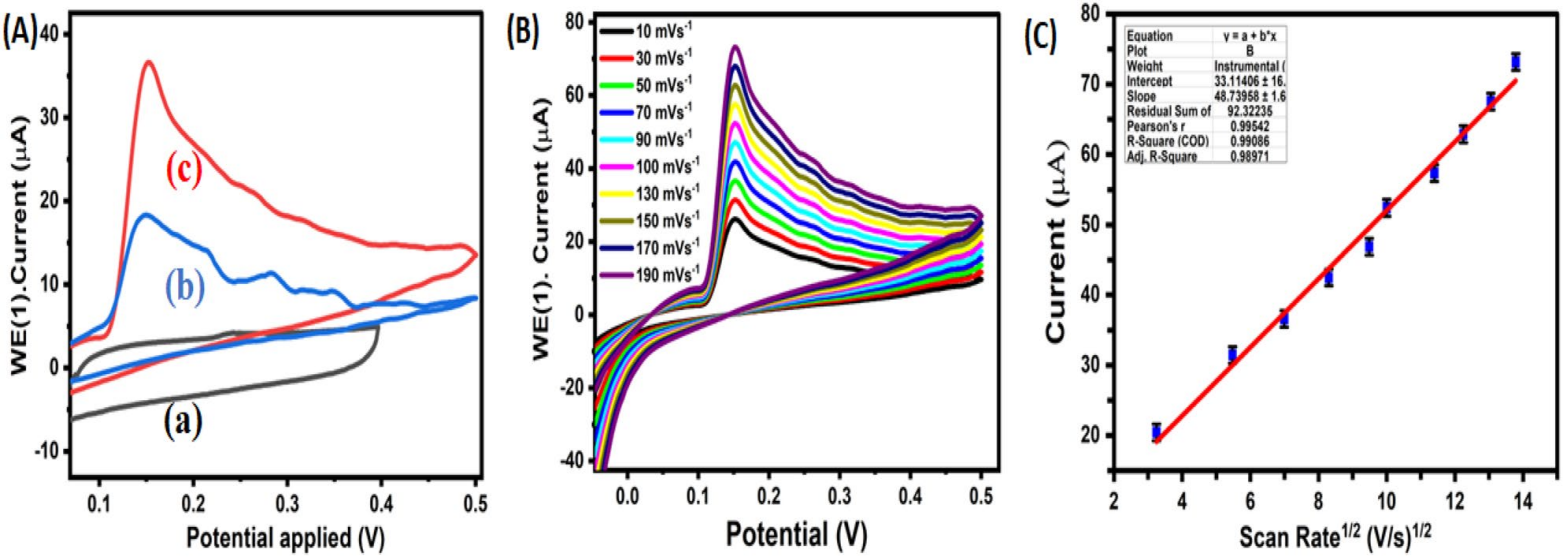
(**A**) Electrochemical oxidation of 0.02 µM Vitamin D3 at (a) GCE, (b) CuO/Ag@n-hexane-GCE and (c) CuO/Ag@lemon extract-GCE electrodes at potential range of 0.1 to 0.5 V at a scan rate of 30 mVs^−1^. (**B**) Effect of scan rate (10 mVs^−1^ ‒ 190 mVs^−1^) on oxidation of 0.02 µM Vitamin D3 at CuO/Ag@lemon extract-GCE electrode. (**C**) Plot of Current (µA) vs scan rate.

Cyclic voltammetry was employed to investigate the electrochemical oxidation of Vitamin D3 at modified electrodes including GCE, CuO/Ag@n-hexane-GCE, and CuO/Ag@lemon extract-GCE. The experiments were conducted in 0.1 M KCl, serving as the supporting electrolyte, with 0.02 µM Vitamin D3. The potential range was set from 0.1 to 0.5 V, and the scan rate was 10 mVs^-1^ (Fig. 6A). The results indicated that all three modified electrodes (GCE, CuO/Ag@hexane extract-GCE, and CuO/Ag@lemon extract-GCE) exhibited a distinct oxidative peak in the forward scan, while no peak was observed in the reverse scan. This observation suggests that the oxidation process of Vitamin D3 at these modified electrodes primarily involves the oxidation of the triene moiety present in Vitamin D3. This is attributed to the electron-rich nature of Vitamin D3, which contains a triene moiety at the fifth, seventh, and tenth positions, along with a hydroxyl group at the third position (19). The Fig. 5. mechanism of electrooxidation of Vitamin D3 involves oxidation of triene moiety.^41–44^ However, among three conjugated double bounds (C8–C19), it is not certain that where the oxidation occurs primarily. Literature reported proposes hydroxylation may occur at C7 and C8 or at C10 and C19 positions^45^. These reported studies strongly suggest oxidation of Vitamin D3 is an irreversible process involves 2 electrons and 2 protons transfer. On the basis of these studies mechanism of electrooxidation of Vitamin D3 can be summarized in Scheme 2.

To determine whether the oxidation process on the CuO/Ag@lemon extract-GCE modified electrode occurred under adsorption or diffusion control, the impact of scan rate on the peak current of Vitamin D3 was investigated. The experiments were conducted in 0.1 M KCl as a supporting electrolyte, with a Vitamin D3 concentration of 0.02 µM, within a potential range of 0.1 to 0.5 V. The scan rate was varied from 10 mVs^−1^ to 190 mVs^−1^ (Fig. 6B). It was observed that as the scan rate increased, the peak currents also increased, and the peak potential shifted in a positive direction. These findings confirmed the diffusion-controlled oxidation of Vitamin D3 at the developed interface (Fig. 6C).

**Figure 7:**
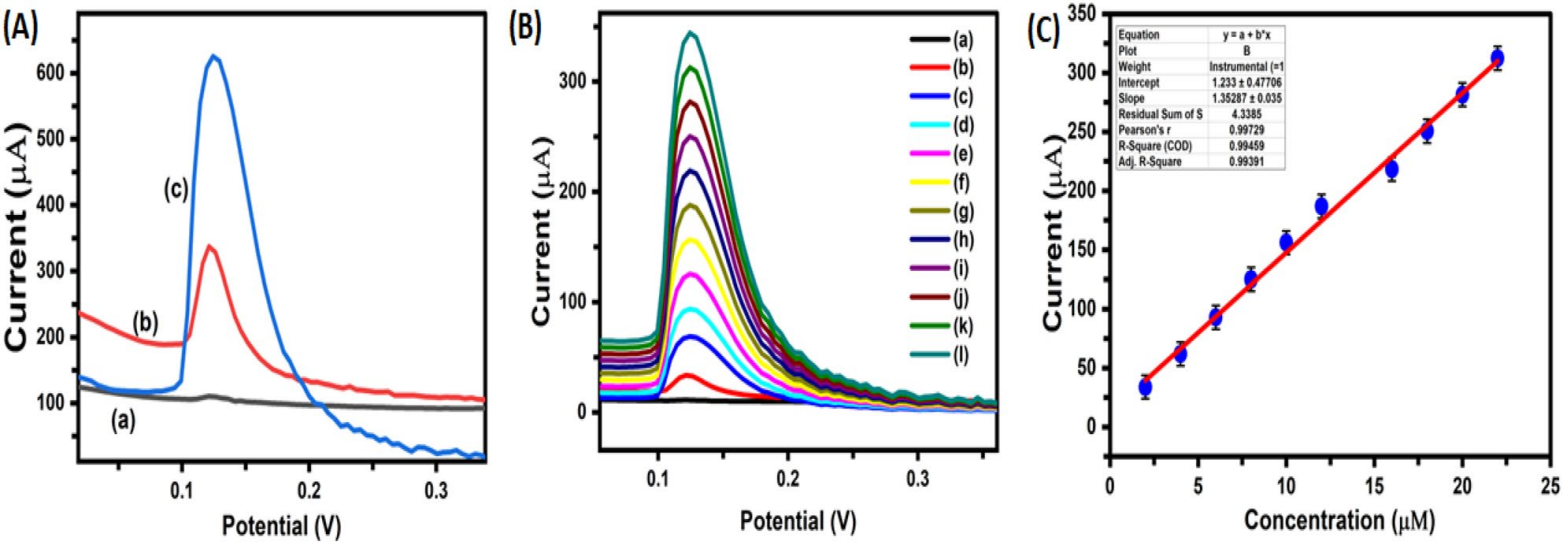
(**A**) Differential pulse voltammograms of Vitamin 36.5 µM D3 at (a) GCE, CuO/Ag@n-hexane − GCE and CuO/Ag@lemon extract-GCE interface (**B**) DPV of different concentrations (0.02–22.5 µM) of Vitamin D3 obtained at CuO/Ag@lemon extract-GCE; (**C**) Curve between peak current vs concentration (µM).

To create main sight about electrocatalytic property of modified electrodes towards electrochemical detection of Vitamin D3, both CV and DPV techniques were employed. Figure 6A shows electrochemical oxidation (CV) of Vitamin D3 at GCE, modified CuO/Ag@n-hexane—GCE, and CuO/Ag@lemon extract—GCE. A broad oxidation peak (0.25 V) was obtained at GCE, whereas less positive potential peak (0.15 V) and two-fold higher oxidation current was obtained at CuO/Ag@hexane − GCE electrode. These results of modified CuO/Ag@hexane − GCE electrode justify an advantage of using bimetallic nanoporous CuO/Ag materials for sensing of Vitamin D3. The reason for higher electrocatalytic activity of CuO/Ag@hexane − GCE modified electrode is attributed to the synergistic effects of bimetallic CuO/Ag nanoporous materials which outperform in comparison to their single-component counterparts by improving current density, lowering over-potential and hence enhancing electron transfer kinetics between electrode–electrolyte interfaces. Moreover, a substantial increase in peak current (fourfold higher) of Vitamin D3 oxidation was observed at CuO/Ag@lemon extract—GCE interface credited to presence of citric acid in lemon extracted CuO/Ag nanoparticles which accelerates the reduction of metal precursors, increases surface area, porosity and aspect ratio and hence leads to higher sensitivity towards detection of Vitamin D3. The electrocatalytic activity of the CuO/Ag@lemon extract-GCE interface was further evaluated using differential pulse voltammetry (DPV), as shown in Fig. 7A. The obtained results from DPV were consistent with those obtained from cyclic voltammetry, indicating similar performance and confirming the electrocatalytic activity of the constructed CuO/Ag@lemon extract-GCE interface.

### Sensitivity of CuO/Ag@lemon extract-GCE sensing platform

To evaluate the electroanalytical performance of the developed CuO/Ag@lemon extract-GCE interface, differential pulse voltammetry (DPV) was employed as a reliable method. DPV was used to assess the linearity of the framework and calculate the limit of detection (LOD) under the optimized conditions. Figure 7 (B) demonstrates DPV curves for electrochemical oxidation of a concentration range of Vitamin D3 at CuO/Ag@lemon extract—GCE interface in 0.1 M of PBS (pH = 7.1). It was observed that anodic peak current for Vitamin D3 oxidation increases linearly with subsequent increase in [Vit. D3] with a slight positive shift. The constructed calibration curve (Ip vs [Vit. D3]) shown in Fig. 7 (C) is apparently linear over a concentration range of (0.02–22.5 µM) with a slope of 0.735 μM μM^−1^ and a correlation coefficient (R2 = 0.994). The detection limit (DL3σ) of 2.62 nM (S/N = 3), where “3σ” represented as standard-deviation from six-blank measurements. The present report and regression data along with the detection limit were very compared with literature values estimated by reported electrochemical measurements (Table 1). The coherent sensitivity of present system is attributed to synergetic effects of bimetallic CuO/Ag nanoporous materials. These synergetic activities of synthesized BMNPs (CuO/Ag) properties acceleration of faster electron transfer kinetics at electrode surface by increasing charge transfer properties. The current electrochemical approach provides numerous benefits when compared to previously reported methods for detecting [Vitamin D3]: (i) simple fabrication process, (ii) long-term stability of modified interface, (iii) high sensitivity (2.62 nM). These analytical characteristics make this method highly suitable for the detection of [Vitamin D3].

**Table 1:** Developed method was compared to previously reported methods for the sensing of Vitamin D3.

Technique	Electrode	Linearity(μM)	LOD(μM)	Reference
SPR	Ab-25OHD/SPE/GCE	12.9–129	2.60	^38^
DPV	BSA/Ab-VD2/CD-CH/ITO	0.025–0.143	0.003	^40^
DPV	SiO2/GO/Ni(OH)2-GCEa	0.25–2.5	0.003	^42^
DPV	GCE	2–400	1.49	^46^
DPV	Au—Pd-GCE	5–50	0.18	^47^
SWV	BDDE	2–200	0.17	^48^
DPV	BSA/Anti-VD/Fe3O4PANnFs/ITO	0.025–0.25	3.11	^49^
DPV	BSA/Ab-VD/AspGd2O3NRs/ITO	0.025–0.25	2.59	^50^
DPV	BSA/anti-25VD3/nCeO2/CC	0.0025–0.51	0.012	^51^
DPV	ASu@MNPs-SPE	0.019 -0.18	0.006	^52^
DPV	**CuO/Ag@lemon extract-GCE**	**0.02–22.5**	**2.62 nM**	**Current method**
Amperometry	**CuO/Ag@lemon extract-GCE**	**0.02 to 0.2**	**2.70 nM**	**Current method**

AuPd-GCE: AuPd modified GCE, BDDE: boron doped diamond electrode, Ab-25OHD/SPE/GCE: CYP27B1/GCE: cytochrome p450 27B1 modified GCE, SiO_2_/GO/Ni(OH)_2_/GCE: silicon dioxide-graphene oxide modified GCE, BSA/Ab-VD_2_/CD-CH/ITO: bovine serum albumin (BSA) ITO glass substrate, ASu@MNPs-SPEcysteamine functionalized magnetic nanoparticles modified screen printed electrode.

Significant values are in bold.

**Figure 8:**
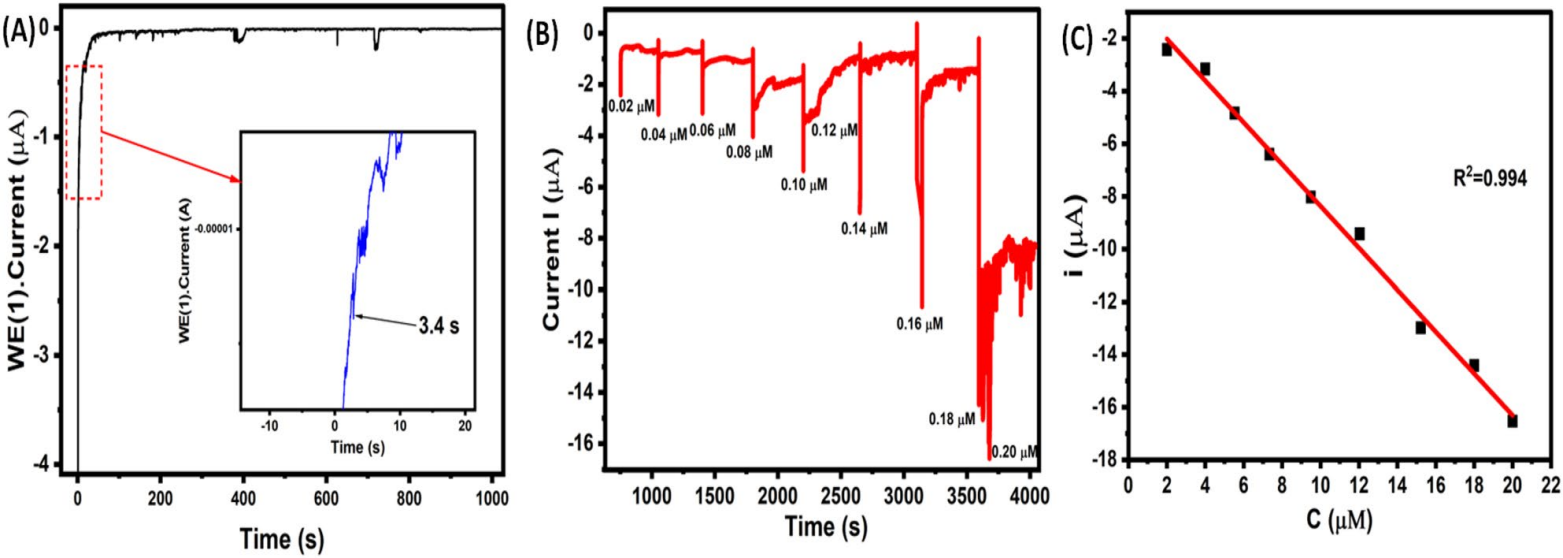
(**A**) Chronoamperograms of 0.02 µM Vitamin D3 at CuO/Ag@lemon extract-GCE modified electrode; Inset view of chronoamperograms of 0.02 µM Vitamin D3 quick response in 3.4 s; (**B**) Chronoamperograms at different concentrations from 0.02 to 23 µM of Vitamin D3 employing CuO/Ag@lemon extract-GCE modified electrode sensor; (**C**) Relationship between concentration and faradaic current.

In order to additional asses the performance of modified CuO/Ag@lemon extract-GCE interface amperometric method was also applied under continuously stirred electrolyte solution at a constant applied potential. As shown in Fig. 8 A, 0.02 µM of Vitamin D3 was injected into constantly stirred 0.1 M KCl electrolyte solution, the CuO/Ag@lemon extract-GCE modified electrode exhibited a rapid amperometric response in 3.4 s and obtained response was constantly stable for more than 1000 s. Further, CuO/Ag@lemon extract-GCE modified electrode was used to examine concentration dependent activity towards determination of Vitamin D3. As shown in Fig. 8 B, the periodical injection of Vitamin D3 from 0.02 µM to 0.2 µM to the continuously stirred 0.1 M KCl electrolyte solution displayed a noticeable amperometric responses indicated that significant sensitivity towards the wide-range detection of Vitamin D3.

The calibration plot was derived from function of Vitamin D3 concentrations vs. amperometric current response that exhibited a linear fit indicating potential sensitivity against wide-range of Vitamin D3 (Fig. 8C). The LOD of developed sensing platform was obtained by using linearity plot from Fig. 8C about 2.70 nM (S/N = 3), where “3σ” represented as standard-deviation from six-blank measurements. The LOD of as fabricated CuO/Ag@lemon extract-GCE modified electrode was indeed comparable with other noble and non-noble metal nanostructures-based Vitamin D3 interface (Table 1).

### Anti-interference ability test, reproducibility, stability, and reusability tests

**Figure 9:**
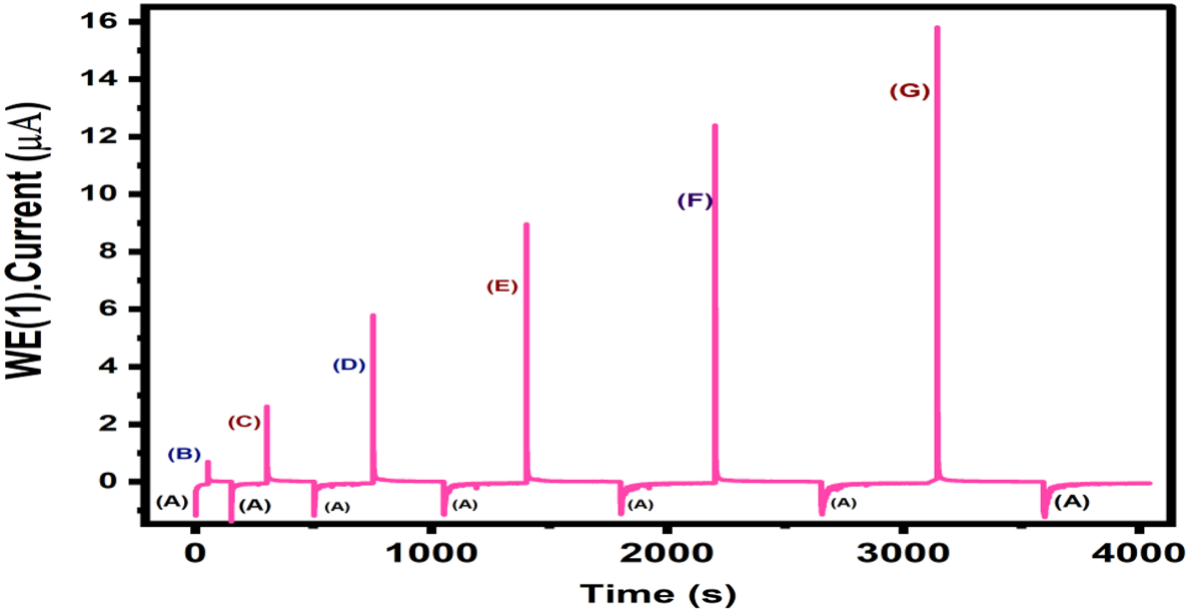
Comparison of the amperometric responses of interferences [Mg^2+^, Ca^2+^, Na^+^, Cl^-^, K^+^ and cholesterol, L-cysteine, uric acid, and glucose] for detection of Vitamin D3.

The specificity of fabricated sensing CuO/Ag@lemon extract-GCE platform was studied by adding substances such as organic compounds viz., cholesterol, L-cysteine, uric acid, and glucose and inorganic ions Na^+^, Mg^2+^, Ca^2+^, Cl^−^, K^+^ for perception of Vitamin D3. The selectivity assessment was conducted by introducing 20 µM of various compounds into an electrochemical cell containing a 0.05 µM of Vitamin D3 solution. Analysis of Fig. 9 demonstrates that the amperometric responses of Vitamin D3 remained largely unaffected by the presence of interfering agents, indicating that the developed interface maintains its robustness even in the presence of these compounds.

The CuO/Ag@lemon extract-GCE modified electrode demonstrated reproducibility and reusability when tested using the amperometric technique under equivalent conditions. The relative standard deviation (RSD) values for reproducibility and reusability were found to be 94% and 82%, respectively, falling within an acceptable range. This confirms the good shelf-life and reproducibility of the fabricated CuO/Ag@lemon extract-GCE interface. Furthermore, the stability of the fabricated interface was evaluated over a period of 30 days, with measurements taken at intervals of 7 days. It was observed that the fabricated interface maintained 92.3% of its initial current response after 25 days and 82.7% after 30 days. These results indicate that the CuO/Ag@lemon extract-GCE platform is deemed suitable shelf life of 25 days.

## Conclusion

In conclusion, Vitamin D3 deficiency has become a prevalent health concern due to indoor lifestyles and inadequate dietary intake. The deficiency has been linked to various health complications, including cardiovascular diseases. Traditional methods used for the synthesis of BMNPs are costly and result in the production of hazardous substances, posing risks to the environment. In order to minimize the potential toxicity in the environment, it is crucial to adopt greener approaches, which are environmentally friendly and safe. Therefore, the present study presents a green, non-toxic, and straightforward technique for synthesizing bimetallic nanoparticles (BMNPs) using citrus lemon extract. Various experimental factors were optimized, including contact time, the concentration of the extract, and metal ion concentration, to achieve the synthesis of CuO/Ag BMNPs. This study presents a novel nanoporous material, CuO/Ag@lemon extract synthesized using the sol–gel method, and characterized using TEM, XRD, SEM and EDX techniques. The electrochemical properties of these bimetallic nanoparticles were investigated using CV, DPV, and amperometry methods for detecting Vitamin D3. The remarkable sensitivity of the current system is imputed to the synergistic effects of the BMNPs material (CuO/Ag), which enhance electron transfer kinetics due to its high conductivity and improved charge transport capabilities. These effects are not achievable with monometallic nanoparticles alone. The proposed method offers advantages of being relatively fast, simple, and cost-effective compared to previously reported techniques used for Vitamin D3 analysis. This method provides a rapid and sensitive platform for detection of Vitamin D3 with minimal interference from other molecules, enabling early diagnosis and treatment of Vitamin D3 deficiency to prevent severe health complications.

## Data Availability

The manuscript includes all the data necessary for the study.
