# Author Correction: Lemon extract supported green synthesis of bimetallic CuO/Ag nanoporous materials for sensitive detection of vitamin D3

**DOI:** 10.1038/s41598-023-49318-4

**Published:** 2023-12-16

**Authors:** Gowhar A. Naikoo, Fay M. Almashali, Fatima A. S. Habis, Mustri Bano, Jahangir Ahmad Rather, Israr U. Hassan, Rayees Ahmad Sheikh, Palanisamy Kannan, Iman M. Alfagih, Murtaza M. Tambuwala

**Affiliations:** 1 Department of Mathematics and Sciences, College of Arts and Applied Sciences, Dhofar University, Salalah, PC 211 Oman; 2 Department of Chemical Engineering, College of Engineering, Dhofar University, Salalah, PC 211 Oman; 3 Department of Chemistry, Govt. Degree College Pulwama, Kashmir, 192301 India; 4 College of Biological, Chemical Sciences and Engineering, Jiaxing University, Jiaxing, 314001 People’s Republic of China; 5 Department of Pharmaceutics, College of Pharmacy, King Saud University, 4545 Riyadh, Saudi Arabia; 6 Lincoln Medical School ‑ Universities of Nottingham and Lincoln, University of Lincoln, Brayford Pool, Lincoln Lincolnshire, LN6 7TS UK

Correction to: *Scientific Reports* 10.1038/s41598-023-46774-w, published online 22 November 2023

In the original version of this Article, Mustri Bano and Jahangir A. Rather were omitted as corresponding authors. Correspondence and requests for materials should also be addressed to mustribano1@gmail.com and jahanneha@gmail.com.

In addition, Fatima A. S. Habis was incorrectly affiliated with ‘Department of Chemistry, Govt. Degree College Pulwama, Kashmir 192301, India.’

The correct Affiliation is listed below:

‘Department of Chemical Engineering, College of Engineering, Dhofar University, Salalah, PC 211, Oman.’

The original Article has been corrected.

